# Care Professionals Manage the Future, Frail Older Persons the Past. Explaining Why Frailty Management in Primary Care Doesn't Always Work

**DOI:** 10.3389/fmed.2020.00489

**Published:** 2020-08-28

**Authors:** Yvonne La Grouw, Duco Bannink, Hein van Hout

**Affiliations:** ^1^Department of Political Science & Public Administration, Faculty of Social Sciences, VU University Amsterdam, Amsterdam, Netherlands; ^2^Departments of General Practice & Medicine of older people, Amsterdam University Medical Centers, Amsterdam Public Health Research Institute, Vrije Universiteit, Amsterdam, Netherlands

**Keywords:** frail older persons, frailty management, professional-patient cooperation, emergency department visits, primary care, dealing with loss, double management challenge, case studies

## Abstract

Frailty management focuses on optimizing the physical and psychological functioning of older people with frail health through early risk identification and intervention in primary care. Such care programs demand a joint effort by primary care professionals and older persons, one in which professionals are expected to promote or facilitate self-management practices and older persons are expected to adhere to the professional advice. It is known that patients and professionals hold different perspectives on frailty, but we know little about how this may affect their cooperation in frailty management. In this article, we therefore study how different perspectives of older persons and their primary care professionals play a role frailty management in practice. Nine cases of frailty management were reconstructed through semi-structured interviews with older persons, their family doctor and practice nurse. Drawing from literature on managing complex problems, we analyzed how “factual” and “normative” orientations played a role in their perspectives. We observe that the perspectives of care professionals and older persons on frailty management were substantially different. Both actors “manage” frailty, but they focus on different aspects of frailty and interestingly, care professionals' rationale is future-oriented whereas older person's rationale past-oriented. Primary care professionals employed practices to manage the medical and social factors of frailty in order to prevent future loss. Older persons employed practices to deal with the psychological, emotional and social aspects of the different types of loss they already experienced, in order to reconcile with loss from the past in the present. These findings raise fundamental questions regarding the different perceptions of and priorities around not only care for frail older people in general, but also implied professional-patient relations and the value of a risk-management approach to care for older people with frail health. The distinction between these perspectives could help care professionals to better respond to older patients' preferences and it could empower older persons to voice preferences and priorities that might not fit within the proposed care program.

## Introduction

A growing number of community-dwelling older people with disabilities and chronic disease experience acute health incidents such as falls, COPD problems and heart failure. This leads to Emergency Department (ED) overcrowding in hospitals (1, 2). In the Netherlands, for example, of the 800,000 ED visits made by older people in 2016, 500,000 could have been prevented according to the Dutch National Institute of Health and Environment (3). Without policy changes, the number of ED visits from older people is expected to grow by 40%—from 800,000 in 2015 to 1,100,000 in 2040 (4).

In the primary-care sector, health risk management is seen as a promising policy response to this cascade of ED visits (5, 6). Specifically, older persons' “frailty” should be “managed” in order to prevent further deterioration, lowering emergency department utilization and chances of hospital readmissions. Scholars have introduced the concept of “frailty” to indicate a status of extreme vulnerability to different types of risks with negative health-related outcomes. Frailty management programs in primary care focus on optimizing the physical and psychological functioning of older people with frail health through early risk identification and intervention. Examples of care programs that can be typified as frailty management are “proactive care” (7, 8), “preventive care” (9), “reablement” (10), and “screening for frailty” (5). Such frailty management programs demand a joint effort by care professionals and older persons, one in which care professionals are expected to promote or facilitate physical activity, a healthy lifestyle and meaningful and pleasurable activities, and older persons are expected to make health optimizing decisions, to adjust their lifestyles and to comply with medication therapy.

Research on the success of frailty management programs shows mixed results (11). Some authors claim that preventive home visit interventions have not been proven effective (12–14), whilst other studies report positive outcomes (7, 15). Healthcare programs that focus on a single disease are more likely to be effective than programs that focus on complex, heterogeneous conditions such as frailty (16). The effectiveness of frailty management programs relates to the intrinsic motivation of frail older people to participate in these programs (17) and to their self-rated health and level of comorbidity (18). Thus, older people with complex health issues, a lower self-rated health and low intrinsic motivation are less likely to benefit from frailty management programs. To better understand why we see this pattern, we need in-depth insight into the lived experiences of frailty management by older persons with frail health and their care professionals.

In this article, we therefore study frailty management as a social process in which care professionals and older people both play a role and have their own perspectives regarding its implementation. By reconstructing the narratives of nine acute health incidents from three different perspectives—i.e., that of older persons who experienced the incident, and that of their family doctors and practice nurse—we have been able to analyze (1) which (different) practices were used to (attempt to) manage frailty, (2) the underlying rationale of these practices, and (3) how these different perspectives could influence the joint effort required by frailty management. Our study contributes to the extant literature by broadening the debate on frailty management by showing how different, coexisting perspectives on frailty management manifest in practice and why we cannot assume that these perspectives can be integrated into a shared perspective of patient and professional. Before discussing our empirics, we first discuss how and why studying frailty management from different perspectives sheds new light on the extant literature.

## Different Perspectives on Frailty Management Practices

Frailty is a complex health problem that is difficult to manage for four reasons. First, because the risk factors that contribute to an older person's frailty can be clinical, functional, behavioral, biological, psychological, emotional and/or social, a broad range of expertise is needed to identify potentially relevant risks (19, 20). Second, because risk factors are often obscure (21) and older persons do not tend to perceive themselves as “frail” (22), they are unlikely to identify when they are at risk themselves. Third, because it is difficult to predict how different risks will interact, it is also difficult to predict how (a combination of) risk factors will develop (23). And fourth, because older people with frail health can suddenly and quickly deteriorate (24) and frailty can fluctuate (25), it is to a certain extent unpredictable.

Scholars have drawn attention to the difference between “medical” and “lay” understandings of frailty and chronic disease management, the former focusing on biomedical risks and the latter on the social consequences of health risks (20, 22, 23, 26). Older persons with frail health and their care professionals have different ideas about frailty, including what constitutes an appropriate care response and what should be prioritized when making care-related decisions (27). The biomedical perspective on frailty remains dominant in chronic disease management programs. Therefore, it is argued that different perspectives on frailty should be acknowledged in care programs (22, 28, 29). Current literature on frailty management deals with these different perspectives by proposing a holistic care program, i.e., by integrating the divergent perspectives of older persons and their care professionals (28, 30, 31). Also, it is thought that the development of a measurable, holistic understanding of frailty should be the highest priority on the frailty research agenda (5, 6). However, different perspectives on frailty cannot easily be integrated in one holistic perspective precisely *because* they are different.

### Factual and Normative Dimensions of Perspectives

The integration of different perspectives on frailty management is intricate due to “factual” and “normative” dimensions of an individual's perspective toward a highly complex problem (32). Perspectives can be understood as a set of ideas about which “facts” need to be known to understand the problem and about what should be done to deal with the problem, the “normative” dimension. These two dimensions are interrelated: a specific factual belief leads to a corresponding normative rationale, and a specific normative orientation influences a person's ideas on which facts are relevant to understand a problem. For example, a doctor may interpret a patient's frailty as a medical problem and propose a medical solution—e.g., a new drug therapy—while an older person may see frailty as a consequence of loneliness and propose a social solution—e.g., chatting with neighbors. This example does *not* show that one person is right and the other is wrong; it shows that their perspectives are *different*. While these are two very different solutions to frailty, both are factually correct justifications in and of themselves: they both solve the problem as it was defined (32). The strategy's justification thus is correct from the individual's own perspective, but does not necessarily comprise or align with the normative preference of the other individual involved in frailty management. This means that we cannot just assume that the integration of two perspectives will produce satisfying results for both care professionals and older persons. Precisely because their preferences are different, the integration of both perspectives is problematic. It is, then, more likely that one preference will “overrule” the other when deciding which intervention to execute.

The different perspectives of older persons with frail health and care professionals may affect the efficacy of the joint effort needed to manage frailty in practice. Effective management of complex problems requires clarity on the factual and normative dimension (33). However, literature shows that frailty management is characterized by very diverse factual and normative understandings. Factually, it is difficult to pin down someone's frail health status and thus normatively, it cannot easily be claimed what will be the best care response. To better understand how frailty management programs work in practice, we must acknowledge that it is a social process in which the different factual and normative orientations of care professionals and older persons toward frailty management may collide. The next step, then, is to characterize the perspectives of care professionals vs. older persons with frail health by identifying which factual and normative orientations they hold. This theoretical lens helps to gain specific insight into how different perspectives might influence the effectiveness of cooperation between professional and older patient.

## Method

### Research Design

A qualitative method fit this study's purpose to better understand the practice of frailty management from the different perspectives of older people and their care professionals, as it allows researchers to interpret people's actions and experiences in terms of the meaning that respondents give them (34). Our study consists of a series of nine in-depth case studies of frail older persons and their care professionals. Case studies are comprehensive examinations of single examples. We used two purposive sampling strategies for case selection, to ensure the sample is relevant to the research question posed. First, one general practice was selected as an *exemplary case*, which means that this practice's care program for frail older persons exemplifies the general trend in primary care toward proactive frailty management. Second, the incident-cases were selected as *critical cases*, which means that these cases show specific characteristics that permit a logical inference about the phenomenon of interest (35, 36). The critical criteria were: (1) the older patient should be in frail health; (2) the older person should have recently experienced an acute health incident; (3) the older person should be involved in a frailty management program; and (4) the care professionals should be experienced with frailty management. These four characteristics create a critical situation: while frailty was “managed,” older persons still experienced an acute health incident. Each case consisted of narrative reconstructions of the acute health incident and frailty management practices as experienced by the older persons who experienced the acute health incident, their family doctor and their practice nurses.

### Research Setting

One general practice, located in a large city in the Netherlands and responsible for the proactive primary care of older people with frail health, participated in our study. The aim of the practice's care was to either maintain or improve the older people's functioning and to prevent avoidable or undesirable acute health incidents.

### Respondents

The first author interviewed nine older patients with frail health, their family doctor and two practice nurses (POH in Dutch). The practice nurses hold a Bachelor degree in nursing and are specialized in elderly care. After an exploratory conversation with the family doctor about the study, the doctor and practice nurses recruited older persons within their care practice for interviews. They searched through their patient files for older persons with frail health who had experienced an acute health incident within the past year. Patients' frail health was determined on the basis of clinical judgment of the family doctor, which is considered to be an accurate frailty instrument (37). They also assessed whether it was psychologically permissible (38) to approach them for this study. Eleven patients were found eligible. They invited the patients either during their routine home visits or by telephone. Older persons who were interested in participating received an informational leaflet about the study. Then, once a patient had read the leaflet and agreed to be contacted for participation, the practice nurse gave the first author the patient's telephone number. The first author phoned the eleven older persons to arrange the interviews. Two patients had told the practice nurse they would participate but ended up changing their minds as they lacked the motivation for a 1 h-interview.

### Procedure

Data was collected in July, August and September of 2017. The first author interviewed the older people at their homes and the care professionals at their general practice. Before every interview, informed consent was discussed. Interview duration ranged from 60 to 105 min. During the interviews with the older persons, respondents' perspectives on (1) the lead up to the incident, (2) the incident itself and (3) the aftermath were discussed, as well as were (4) their experiences with care, ownership of care decisions, quality of life, their living situations and their daily lives. We used the topics of the validated Patient Assessment Integrated Elderly Care (PAIEC) (39) for the fourth part of the interviews. This assessment covers three themes: patient activation and contextual information; goal setting and problem solving; and coordination and follow-up. At this time, permission was also requested to discuss the incident (prior to the interviews) with the respondents' family doctor and practice nurse. While interviewing the care professionals, respondents were similarly asked about their perspectives on and experiences with frailty management vis-à-vis their patients' incidents, including (1) the lead up, (2) the incidents themselves, and (3) the aftermath, as well as about (4) their general ideas on frailty management and each older person's quality of life and ownership in care decisions. The fourth part of the interview was based upon the PAIEC. As we wanted to understand the lived experiences of our respondents, the topics were addressed in a semi-structured way. The respondents were asked to explain their thoughts about the topics and give examples. In these examples, respondents raised other topics that they found relevant in the light of the interview. The interview guides are included in the Supplementary Material. All interviews were audio recorded with permission, transcribed verbatim and made anonymous.

### Data Analysis

We used a narrative analysis strategy. Narratives are stories that people tell about events they have experienced and their evaluative impressions of these experiences (35, 40). Analyzing these narratives enables researchers to interpret people's rationale for their actions. Narrative analysis was done by the first author in two ways: thematically and structurally (40). Thematic analysis meant that she analyzed the content of the interviews by focusing both on “what” was said by respondents and on recurrent topics. Structural analysis implied that the first author looked at “how” respondents told their stories by focusing on narrative structures. The first author's first round of analysis in MAXQDA resulted in clusters of open and axial codes on both themes and narrative structure. The thematic-narrative focus led to the identification of respondents' (different) frailty management practices. The older persons felt uncomfortable with the PAIEC-questions in the interviews, explaining the interviewer that these questions did not make sense to them. In their explanations, older persons emphasized other ways of coping with their frailty. This response showed that the older persons did not experience the proactive, planned approach of frailty management programs as making sense in their lives. These responses informed us to look beyond the proactive paradigm and pay close attention to how the respondents prioritized different matters in managing their frailty on a daily basis. The structural-narrative focus identified different orientations of time in the respondents' stories.

Credibility is established by (1) ensuring that the research is carried out according to the principles of good practice, and (2) checking whether the researchers have understood the respondents correctly, so-called member validation [(34), p. 384]. To ensure the credibility of the first author's interpretations, the transcripts were divided among three other researchers (second author, third author and fourth researcher) who each analyzed three cases. Together, the four researchers then discussed and compared their interpretations of the themes and narrative structures they had identified between the nine cases and the relations between them. Next, the first author selectively coded the identified thematic and narrative codes using the theoretical concepts of “factual dimension” and “normative dimension” to characterize the different perspectives on frailty management. Saturation was reached on eight of the nine cases based on the richness of the data and an overall sense of recurrent themes (41). At this point, new cases did no longer suggest new dimensions to the findings, nor was contrary evidence found for the developed theoretical insights (34). As a last analytical step, the first author brought the perspectives “into dialogue” (35): she looked for (dis)continuities, identified tensions between them and reasoned how these perspectives may relate and interconnect, both within and between the cases. Through the iterative process of open, axial and theoretical coding, as well as through revision and discussion, we sought to be rigorous in our analysis. As a form of member validation, the second aspect of credibility, a summary of the findings in the form of a popular article was sent to every participant in the study, the first author discussed the results in a focus group with representatives from a frail older people network of the city, and the first author presented the results to family doctors and practice nurses from other practices working in the same city within in the field of proactive care for older persons with frail health.

### Ethical Considerations

The study was cleared by the Medical Ethical Review Committee of the VUmc Medical Center Amsterdam.

## Findings

In total, nine community-dwelling, frail older persons within an age range of 79–94 years (mean: 86.8; SD: 5.57), one family doctor and two practice nurses were interviewed to reflect on the nine different cases of acute health incidents that had led each older person to an ED visit (see Tables 1, 2). Narrative reconstructions from these three different perspectives showed how frailty management was experienced in very different ways. Below, we first describe the care professionals' perspectives on the frailty management practices used in the lead up to the acute health incidents. Second, we describe the older person's perspectives on the same matters. Third and lastly, we discuss how these perspectives interact in practice.

**Table 1:** Overview of the nine acute health incident cases.

	**Overview cases acute health incidents**	**Interviewed respondents**
Case 1	Fall incident	Older person 1, family doctor, practice nurse 1
Case 2	COPD suffocation incident	Older person 2, family doctor, practice nurse 1
Case 3	Fall incident	Older person 3, family doctor, practice nurse 2
Case 4	Fall incident	Older person 4, family doctor, practice nurse 2
Case 5	Fall incident	Older person 5, family doctor, practice nurse 1
Case 6	Fall incident	Older person 6, family doctor, practice nurse 2
Case 7	Fall incident	Older person 7, family doctor, practice nurse 1
Case 8	Fall incident	Older person 8, family doctor, practice nurse 1
Case 9	High blood pressure and fall incident	Older person 9, family doctor, practice nurse 1

**Table 2:** Characteristics older persons with frail health in cases.

	**Numbers**
Age	75–79 (*n* = 1)
	80–84 (*n* = 3)
	85–89 (*n* = 1)
	90–94 (*n* = 4)
Sex	Male (*n* = 2)
	Female (*n* = 7)
Marital status	Married (*n* = 2)
	Widowed (*n* = 7)
Living situation	Living alone, apartment (*n* = 5)
	Living alone, senior's apartment (*n* = 2)
	Living with partner, apartment (*n* = 1)
	Living with partner, townhouse (*n* = 1)

### Care Professionals' Frailty Management Practices: Case Management

The interviews with care professionals reflected a narrative of case management. When care professionals were asked to reflect on an acute health incident, they responded by talking about the frailty management practices they had used prior to the incident: summarizing symptoms, collecting information from different sources and estimating the effects of this information. In other words, care professionals' frailty management practices involved *making a case*. Older persons with frail health were seen as “cases” with an eye on the future: what could happen in the future and what should be done today to prevent adverse outcomes? Care professionals used a staccato and distanced speaking style, referring to patients as “it”—a case—and also to care providers who execute a care plan as “it”—a mechanism for managing a(n aspect of the) patient's frailty: “There is a senior care plan *in it* [in the “case”], and they come once a week, also to—well you could say—to talk to him, that is it. But *it* [the “care plan”] showers him as well” (practice nurse, case 1). This style could underscore the idea that care professionals see their patients' frailty as a collection of different aspects that need to be checked and managed.

To manage their cases, the care professionals described to employ different practices. We extracted four “case management strategies” from the data: *proactive monitoring, proactive planning, multidisciplinary collaboration* and *tightening of the strings*. These strategies can be interpreted as mechanisms for frailty management in their case management narrative. First, *proactive monitoring* is an important task of practice nurses: “She also has someone who visits her at home for her COPD now, so we're monitoring everything now” (practice nurse, case 2). As seen in our study, practice nurses generally visited their older patients with frail health once every 3 months to screen them for frailty risk factors:

There are a number of things we do can objectively: is there a possible urinary tract infection? We check for that. Is there something odd with their blood pressure? Is there something with, well, we also check for diabetes at the diabetics check. We look at their general sense of dizziness too, if they're walking strangely or also when people suddenly lose weight (practice nurse, case 4).

Second, *proactive planning* is described by the care professionals as the following step in their frailty management efforts. Instead of only being responsive to older people's care needs, care professionals set proactive goals for the improvement of their patient's current, frail situation — to make them stronger, healthier and/or more resilient. The following quote shows a description of proactive planning practice, narrated in an abstract, case management style:

Then we make a few plans or goals and then we first go tackle those, and then eh—look, and then we want *him* [patient is seen as a “case”] to *come back* next time [the case will come back on the agenda of the next multidisciplinary consultation], like, what have we achieved at that point? (practice nurse, case 1).

Third, the care professionals stressed the importance of *multidisciplinary collaboration* for frailty management, because it enables them to generate an understanding of the high complexity of someone's frailty through the use of different opinions and knowledge. “I must say, the physical therapist is doing so much with people, such good work. Strengthening their muscles, reducing their risk of falling, providing insight into their own movement” (practice nurse, case 4).

We look indeed at the different opinions [of other care professionals, in multidisciplinary consultation], with each other, like hmm, like okay, we need to look at this as well. Who is this person [patient], what does that [social] system look like—eh, the family doctor knows that exactly, he has known [this patient] for a very long time of course (practice nurse, case 1).

Fourth, in response to an acute health incident, care professionals described to *tighten the strings* and controlling for possible contributing factors:

It turned out, I think, that it [ED admission] was necessary, you could say. So whether we had seen it [the incident] coming, I don't know. It was a mix of a lot of things. […] Later it turned out that she also had a form of, I thought COPD or something, and that plus the fact that her inhaler wasn't compatible with what she used before. So when she was here [at the general practice], we took steps to improve that (practice nurse, case 2).

These responses show how the interviewed care professionals' “factual orientation” sees frailty as a collection of medical and social risk “facts” that needed to be identified, measured, step-wise addressed and controlled. This factual understanding of frailty is related to a “normative orientation” on preventing future loss. Care professionals employ these structured, planned practices because future incidents need to be prevented and, should they still happen, at least professionals need to ensure they cannot be blamed: they have done everything possible within the limits of their knowledge and capacity. All in all, in terms of frailty management, these findings can be summarized as a perspective of future-oriented case management (Table 3).

**Table 3:** Care professional perspective.

**Case management practices**	**Examples**	**Factual orientation**	**Normative orientation**
Proactive monitoring	Blood pressure screenings, weigh-ins, the timely detection of ailments.	Medical and social facts that construct frailty need to be identified and measured.	Future oriented: preventing future loss.
Proactive planning	Making a care plan with future goals, e.g., improving muscle strength, lowering blood pressure.	Medical and social facts that construct frailty need to be predicted and translated a step-wise plan to respond to future risks.	Future oriented: preventing future loss.
Multidisciplinary collaboration	Organizing multidisciplinary consultations, sharing information and tasks.	Facts from a wide variety of disciplines that construct frailty need to be detected, identified and acted upon.	Future oriented: ensuring a holistic approach to be able to prevent future loss.
Tightening the strings	Changing medications, increasing check-up frequency, including care professionals from different disciplines.	Medical and social facts that construct frailty need to be controlled.	Future oriented: preventing future loss.

### Older Persons' Frailty Management Practices: Dealing With Loss

The interviews with older persons with frail health reveal a narrative of dealing with loss. When the older persons were asked to reflect on their own acute health incidents and its lead up, each responded hesitantly. During the interviews, the older persons reported having little recollection of the specifics surrounding the lead-up to the incident and thus limited potential explanations as to why it happened. To them it “just happened” and they felt unable to identify clear signals or causes, whether before the incident or in retrospect:

Before I knew it I was lying on the floor! I came out of the mall and then, actually, I wanted to close my jacket. Yes and then, whether I got dizzy I can't remember either. But then I… I don't know! (…) You can't prevent it because there I was, lying there. So unexpectedly (older person, case 8).

When the older persons were asked about their thoughts on preventing future incidents, including a care plan and its goals, they responded with sighs, silence or awkwardness. The majority of the older persons vocalized not finding it useful to think about such concerns. As they explained why, they turned the topic of conversation to loss: when you have already experienced a lot of loss in life, you lose your belief in the usefulness of prevention and try to avoid thinking about new potential losses in the future. They expressed that the experience of preparing for future risk felt alienating, confrontational and sometimes painful. As such, the interviewed older persons perceived their frailty as a state of loss: “Setting goals? That's something for young people, but at my age…” (older person, case 5).

Instead of making plans for future deterioration, the interviewed older persons focused on reconciling past loss in the present. We identified four ways they dealt with these feelings of loss: *by accepting ailments as a part of daily life, by putting their own situation in perspective, by living day to day and trying to keep doing what they had done before*, and *by grieving their losses*. First, because the older persons had lost many of their physical capacities and were confronted with ailments and pain on a daily basis, *accepting ailments as a part of daily life* was experienced as key to “living a little”:

I already had pain of course. I still had pain and, and I didn't know what [it was]. (…) You could say yes, if you're older you should think about—but my husband is also older, what are you supposed to think about [in terms of prevention]? Yes, you can think about all sorts of things, but I still want to live a little. (…) Should you then—once you've passed uh, 70 or something, or when you're getting close to 80—keep on thinking about what could go wrong? Should I already start organizing in-home nursing care? No! (older person, case 4).

And that one [doctor] said, “Well, it is quite possible that you broke something, but I can't see that now. But if it all gets worse, then you'll have to go to the hospital in an ambulance.” I say, “Okay, I'll see what happens then.” (…) I couldn't care less. I'll see what happens again the next day (older person, case 7).

Second, the older persons with frail health frequently *put their own situations in perspective* by comparing themselves to others who were worse off and by downsizing their own problems. They compared themselves with other older people who faced (combinations of) dementia, cancer, disability, depression, and loneliness, but also to younger people (e.g., their children) who they perceived as vulnerable.

I was never sick—well, I had that [cancer] radiation. But I was lucky. I had a friend. I stood with him at the hospital desk to register and he… He came out of the elevator. He stood next to me. After 2 months he was already gone. And I hear this a lot from acquaintances and friends (older person, case 9).

Third, all respondents stressed the importance of *trying to keep doing what they had done before*. Thinking about everything that could go wrong in the future felt, as they reported, like it took attention away from what they were still able to do—which would threaten not only their sense of identity, but also their quality of and purpose in life.

When I go out with my friend we walk too far and I'm aware of that. But I don't want to ruin it so I just keep on walking. But then the next day it does hurt for a while. (…) I could do anything, back when I was healthy. [silence] Yes, age of course plays a role. I get tired. And I'm not used to that. I mean, eh, I've been playing tennis from my 25th until my 85th. I used to live in an area where we went ice skating during the winter. And then you received a medal—I skated 600 km. So I mean, I'm strong. I've never had anything and then suddenly. (…) You slip into it. And that is tough. You have to accept it. That's difficult (older person, case 9).

For some respondents, *trying to keep doing what they had done before* meant not letting themselves slip into a depression. One older person told the interviewer about the great loss of her two children and husband, for which her (ongoing) coping mechanism was “just staying put”: “In the short time that I have left, I'll just stay put. I'll never do anything crazy, absolutely not. And you never know when it's time, and yes—I have had the longest time” (older person, case 3).

Lastly, respondents told stories about the role of *grief* in their daily lives. They described grieving their lost loved ones (partners, family, friends and children), their physical capacities (walking, traveling, housekeeping, sports, knitting, gardening, concert going, etc.), their memory (forgetting their medicine, finances, administration, or even who is visiting), their personal property (the house in which their children grew up, material items with emotional value, e.g., records, encyclopedias, furniture, cars, their garden) and their purpose in life (their job, hobbies, ability to help others, travel,

I don't dare say it. [Laughs nervously, then silence.] For me, life has no use anymore. […] If you look at my activities: embroidery, painting over there [points at a painting in the living room], making cards and knitting piles of sweaters and cardigans and whatever they all wear. All those things have become impossible. I get up at 8 am, then I go sit here and eat my yogurt and well. Well, it's not 10 am yet. And in a while, you [the interviewer] will be gone and then I'll go to the square. And then I'll walk across the square, hop by the pharmacy to drop off a prescription for my neighbor. Well, that's about it. And then I'll sit here again. And back in the day, it was reading and socializing and you name it. Everything you did, it's no longer possible. And. All your friends pass away (older person, case 6).

Such responses show how the interviewed older persons' “factual orientation” sees frailty as loss of medical, social and emotional “facts” that needs to be ignored, toned down, protected and acknowledged. This factual understanding of frailty is grounded in a “normative orientation” of reconciling with loss from the past in the present. The older persons viewed the ignorance, downplaying, protection and acknowledgment of loss as the most relevant understanding of frailty because it shifts their attention to their normative priority: reconciling with loss and maintaining their identities and lifestyles from the past in the present. All in all, these findings can be summarized as a past-and-present oriented dealing-with-loss type of frailty management (Table 4).

**Table 4:** Older person perspective.

**Dealing-with-loss practices**	**Examples**	**Factual orientation**	**Normative orientation**
Accepting ailments as a part of daily life	Ignoring pain or ailments; avoiding thinking about future health goals.	Medical facts that construct frailty need to be ignored, e.g., deliberately ignoring potential signs of physical loss.	Past-and-present oriented: reconciling with loss of physical capacities from the past in the present.
Putting their own situation in perspective	Comparing self to others who are worse off (e.g., sister with dementia, daughter in poor health, late friends).	Social and emotional facts need to be placed in perspective, e.g., toning down their own daily experience of loss.	Past-and-present: reconciling with all types of loss, e.g., physical capacities, social network, living situation.
Living day by day and trying to continue doing what they used to do	Not using a walker; providing informal care to partner; cooking despite becoming blind.	Medical and social facts that construct frailty need to be valued considered in relation to identity threats.	Past-and-present-oriented: maintaining their identities and lifestyles as built in the past; maintaining a feeling of self and purpose in life.
Grieving	Reminiscing about lost loved ones, lost social relations, meaningful activities that are no longer possible, e.g., traveling, reading, ice skating.	Social and emotional facts that construct frailty need explicit acknowledgment and attention, e.g., on one's daily experience with loss.	Past-and-present-oriented: being attentive to present grief caused by past loss.

### Care Professionals' and Patients' Perspectives “in Dialogue”: Dilemmas in Normative Preferences

The existence of these two frailty management perspectives shows how managing (an interpretation of) frailty is different for care professionals vs. older persons themselves. To understand the tension between these two perspectives, we put these perspective “into dialogue” (35): identifying (dis)continuities and tensions between the perspectives, and reasoning how these perspectives can relate and interconnect. When we compare Tables 3, 4, we see that both tried to get a grip on reality through sense making (of the patient's frail health situation), but also that both told different narratives in order to do so. Whereas, care professionals were focused on controlling frailty by checking as many frailty risk-factor “boxes” as possible—with an initial focus on physical well-being and a normative preference for the prevention of future loss—older persons predominantly tried to shift their attention away from their complex health issues while concentrating on the psychological, emotional and social challenges of being frail and displaying a normative preference for reconciling with past loss.

These differences indicate different priorities, but do not imply that care professionals and older persons do not acknowledge and honor each other's perspectives in practice. In fact, care professionals showed a conspicuous understanding of and empathy for their older patients' initial discomfort to, e.g., adapting their lifestyles or accepting a care intervention for preventative reasons. In response to this discomfort, care professionals adapted their implementation of an intervention to “the older person's own pace,” and emphasized that older persons should feel in charge of their own lives. Likewise, the interviewed older persons showed respect for their care professionals' risk-minimizing orientation by, e.g., being extra careful to take medication following a procedure, or “not doing stupid things” such as washing windows on a ladder.

This process—of acknowledging and honoring the other's perspective—worked out well for both parties as long as it did not (directly) interfere with their own normative preferences. Tensions arose both when the older persons felt that their practices for reconciling past losses were threatened by their care professionals' attempts to minimize risk factors and when care professionals felt that their older patient's lifestyle choices posed a threat to their aim of risk management. In two cases (4, 6), the older patients were determined to continue cooking their own meals—because they enjoyed it and stopping would imply a loss of identity and independence. A practice nurse explained how she had suggested that one of her older patients switch from a gas stove to an electric one, but the patient “was not open to it,” which worried her:

Why not go for peace of mind? I find it scary. And that's the tricky part, because it's a trade-off people make for themselves; between doing what you've always done because you know that on autopilot and therefore it feels safe. That's the choice she's made in this case' (practice nurse, case 6).

Another example concerns physical therapy. The interviewed care professionals believed in the effect of preventive physical therapy to reduce the risk of falling. One older respondent, who did intensive physical therapy as part of her revalidation after a fall, enjoyed the exercises but quit when she moved back home because she felt unable to combine weekly sessions with the informal care she provided for her husband (case 8). She prioritized what she was used to doing—taking care of her husband—over managing potential future risks.

Lastly, it could be argued that these tensions show how frailty management may entail a *loss dilemma* for older people. When they do not cooperate with their care professionals' practices, the older persons inherently accept risks of acute health incidents in the future. When they do cooperate, however, their care professionals' practices may interfere with their own attempts to reconcile with their losses from the past. Both outcomes result, then—when the older person values both risk management *and* dealing with loss—in a new loss.

## Discussion

While scholars currently see the development of a measurable, holistic understanding of frailty management (5, 6), and by extension its preventive ability to reduce ED-visits (1, 2), as the most relevant step toward improving care for older patients with frail health, our study shows how the difficulty of frailty management in practice is not solved by solely generating more knowledge on frailty as a (bodily) phenomenon. Our study shows the importance of the social aspect of the frailty management process and the roles and attitudes of care professionals and older adults herein. A deeper understanding of this social process helps to understand why frailty management does not always work and could contribute to frailty management in practice. In an effort to widen the scope of what frailty management entails, we contribute to the extant literature by addressing fundamental issues about (1) different, co-existing perspectives on frailty management, (2) implied patient-doctor relations concerning health risk management, and (3) the desirability of using frailty management as a form of risk management in all cases.

First, we have demonstrated how frailty management involves two disparate perspectives: the future-oriented, case-management perspective of care professionals and the past-and-present oriented, dealing-with-loss perspective of older patients. Because each follows its own factual and normative understanding of frailty management (32, 33), the incongruity of these perspectives does not disappear in the face of cooperative frailty management. Consequently, their co-existence influences how frailty is actually managed in practice. Both deal in their own way with the older person's frail health status but prioritize things differently. This knowledge could inform the gap in the literature on why frailty management programs are less likely to work for older persons with lower self-rated health (18), complex health issues (16) and low intrinsic motivation to participate in these programs (17): older people with frail health who experienced an acute health incident see their uncertain future as something that is less relevant to focus on than their past and present, and thus do not cooperate with a future-oriented care program. Our study shows that it is relevant to look at the *content* of the intrinsic motivation of older people with frail health—what intrinsically motivates them in dealing with their frail health—instead the extent to which they are motivated to participate in a frailty management program. While previous scholars have also indicated a difference in perspective between care professionals and older persons (20, 22, 26, 28–30), they did not structurally characterize these differences on factual and normative orientations toward frailty, or analyze the consequences of different orientations for cooperation between professional and patient in practice. Our results show how professionals' and patients' perspectives are not “just” different, they also seem related to their positions. Care professionals are in a position to manage cases and are responsible for providing good care: they look to the future. Older persons have experienced severe loss and are aware that they are in the last phase of their lives: they focus on the past. In Western societies, people are generally expected to reason in a future-oriented way regarding their health but, for some people, living an enjoyable life today is more pragmatic than investing in a “mythical” future health (42). Our study shows how this is specifically relevant to older people with frail health, whose future by itself could literally be a myth.

Second, our findings seem to exemplify Foucauldian thought on risk-management practices in healthcare. In this research tradition, the term “case management” is used for clinical practices that use individualistic and epidemiological data sources to govern individuals who are deemed threatening or disruptive to the social order. Through various discourse, strategies and practices, distinctions are made between those (1) with greater or lesser risk of illness, (2) who are more or less utilizable or productive, and (3) who are more or less able to be coached toward the health standards set by clinical experts (43, 44). In our case, overcrowded EDs could be understood as a Foucauldian threat, which calls for case management practices that force patients to become “docile bodies” who manage themselves in line with specific policies (43). Our findings show, however, how this view neglects human agency and the possibility of self-management that is not governed by a clinical logic. Indeed, while patients are usually regarded as passive, recipient actors in care relations (45), our study shows how the lived experience of “managed” older persons is richer than that of being a mere “docile body.” Older persons were willing to cooperate with care professionals and their practices as long as doing so did not interfere with their normative, past-focused preferences. When case management interventions did threaten older persons' priorities, they showed self-managing capacities according one's own personal preferences—especially when case management practices were experienced as a potential new loss, e.g., refusing to use a walker in order to maintain an able-bodied identity.

Third, given our observation that older people prioritized dealing with loss over managing future risks, our findings question the desirability of a future-oriented risk management perspective in all cases. Today's primary care programs for older people with frail health strongly steer toward proactively optimizing health (8, 10, 46). These programs have potential to create positive outcomes—when patients are intrinsically motivated and share a future-oriented perspective—but we should not assume that it matches with all patients' priorities and preferences. Care programs often try to motivate chronic patients for risk-avoiding self-management by sharing information on health risks (23), but our results suggest that it is unlikely that more risk knowledge will make older persons' shift from focusing on loss toward prioritizing future care goals. Instead, frailty management is a dialogue in which care professionals and older people need to acknowledge the other's different understandings of autonomy and self-management to deal with frailty. When this difference in perspective is not acknowledged and voiced, the risk could arise that the older person's perspective is missed or the older person does not work along with the frailty management program—which can be overt (telling the professional to disagree) but also covert (agreeing to use a walker but not using it practice). Also, the distinction between a future-oriented care perspective and past-oriented loss perspective could help older persons in making sense of the rationale of a proposed care intervention and why they could feel uncomfortable with it. This could empower older persons with frail health to voice preferences and priorities that might not fit within the care approach as proposed by care professionals.

A care approach that does not focus on health optimization is palliative care. Family doctors' awareness of palliative care needs often arises gradually and relatively late in disease trajectories of their oldest patients. The key point for family doctors to start with a palliative trajectory is the diagnosis of a life-threatening illness (47). This key point complicates the integration of a palliative trajectory in frailty management because frailty is not an acute threat. Older people's frail health rather develops on a subthreshold level and fluctuates in severity (21, 25). As a consequence, we cannot speak of a relatively strict demarcation between the preventive and palliative phase of frail elderly care. The family doctor is, therefore, dependent on the older patient to determine this transition from preventive to palliative per individual case. This underlines our argument that frailty management benefits from a dialogue between professional and patient in which different perspectives are acknowledged.

Our study also has important implications for future research: it underscores the usefulness of investigating and honoring different perspectives when it comes to the embodiment of frailty management. In the future, scholars may want to test to what extent this perspective can be generalized to a broader population of older persons with frail health. Also, it would be interesting to explore how care for older people with frail health may be organized according to various factual and normative orientations instead of just one. The challenge is not to “solve” frailty's factual and normative diversity—since this is, in fact, inherent to the concept of “frailty” itself—the challenge is to find ways to build health programs that are attentive to these two dimensions and to the lived experiences of the actors involved; e.g., programs in which care professionals are still future-oriented and able to manage cases, but with a different care goal. Instead of targeting to reducing health risks, professionals could direct their case management practices toward achieving the highest possible quality of (geriatric) life as defined by the older persons themselves. Such a switch in focus could still allow care professionals to manage cases while better accommodating for an older person's need to deal with loss.

### Strengths and Limitations

Thanks to the design of our research—i.e., zooming in on nine different cases of acute health incidents according to three different actors—the strength of this study is the depth with which we have been able to depict the lived experiences, practices and underlying factual and normative orientations of participants. Health incidents are often analyzed by identifying the root cause and addressing it as a single issue or by examining the healthcare system and imagining how the weaknesses found in the procedures and services may have led to the undesirable outcome (48). Neither approach, however, allows for the coexistence of different perspectives on the healthcare problem. Our identification and analysis of two competing perspectives does allow for different, coexisting “diagnoses” of frailty management, which provides a new analytical perspective on the root of healthcare problems. While incident analyses tend to focus on single events, our study of a series of cases provided insight into the structural themes that help explain the observed practices (48). Next, since patients are rarely interviewed after incidents, the patients' perspectives provided an exclusive look into the experienced healthcare system (38). The qualitative methodology chosen allowed for the identification of the topic of loss. This topic was not included in the topic list but was recurrently addressed by the older persons themselves.

A limitation of the study is the fact that we only included professionals and patients within one general practice. The inclusion of more practices could have offered a different practice of frailty management. Though, the selected general practice is an exemplary case because it has worked for years with a proactive care program for frail older adults, which is in line with the general trend toward proactive frailty management in primary care. The fact that all patients had the same family doctor and one of the two practice nurses is a selection bias of the study. The older persons could have responded in certain ways because they receive care from these specific professionals and because they live in a specific area. As we only included patients from this general practice who had experienced an incident within the past year and were willing to participate, we could only select a relatively small number of participants. Due to the older persons' frailty, their perspectives were apt to be different on a better or worse day. Nonetheless, as we found similar patterns among the nine cases, we expect that the characteristics identified in respondents' perspectives are representative of their general state of mind. Moreover, our choice to select participants based on an incident history and to structure the interviews around these events could invoke the belief that frailty management itself is unproductive, because the incident they experienced had not been prevented. The quality of care practitioners' responses could have been influenced by an impulse to defend their frailty management practices and avoid blame for the incidents. Lastly, seven out of nine acute health incidents concerned a fall. This can be explained by the fact that community-dwelling older people with frail health are highly likely to experience a future fall (49), so it is a common acute health incident for this specific patient population. Different acute health incidents may have led to different results, although the 2 other incident cases did not deviate from the fall-cases.

Due to the two chosen purposive sampling strategies, our study offers starting points for further theory development. Because we found future vs. past-and-present perspectives in this exemplary case, these perspectives could also be relevant for other general practices working with similar care programs. We studied a general practice experienced with proactive, preventive care for older persons with frail health, so it could be the case that general practices without an explicit focus on future goals also have a group of patients with a past-and-present perspective. Because we selected on critical cases, we knew that the selected older persons did not just live with the threat of future problems; they had already experienced at least one serious health incident. As the selected older persons—with very frail health—showed autonomy in determining their own health priorities and shifting their attention away from frailty managing practices, it could imply that older adults who are in better health are able to show as much if not more autonomy in deciding whether or not to cooperate with frailty management practices.

## Conclusion

In this article, we have studied the different perspectives of care professionals and frail older persons on frailty management after a recent acute health incident, why a difference in perspectives exists and how this difference influences cooperation between professional and patient in frailty management programs. We observe that perspectives of primary care professionals and older persons with frail health are fundamentally different. Healthcare professionals focus on the future through case management practices, while older persons with frail health avoid to look in the future and prioritize practices to deal with loss from the past. Our findings may explain why frailty management does not always work: older people with frail health who experienced an acute health incident view future risk management practices as less relevant than reconciling with loss from their past in the present and thus do not cooperate with frailty management programs. While holistic conceptualizations of frailty are often seen as a solution to different perspectives on frailty, we demonstrate that in practice different perspectives are not integrated: they coexist. Cooperation between care professional and patient can be enhanced by approaching it as a dialogue in which both perspectives are acknowledged as meaningful and valuable. Acknowledging both perspectives creates space for a past-and-present orientations to frailty management next to a future-oriented perspective. This way, care professionals could respond better to older person's needs and older persons with frail health could feel empowered to voice preferences that might not fit within the proposed care program.

## Data Availability Statement

The datasets generated for this study will not be made publicly available because of the privacy of the respondents. Requests to access the datasets should be directed to the corresponding author.

## Ethics Statement

The studies involving human participants were reviewed and approved by Medical Ethical Review Committee of the VU Medical Center Amsterdam. Written informed consent for participation was not required for this study in accordance with the national legislation and the institutional requirements.

## Author Contributions

YL: conceptualization, software, formal analysis, methodology, investigation, writing—original draft and project administration. DB: writing—review & editing and supervision. HH: methodology, writing—review & editing, supervision, project administration, and funding acquisition. All authors contributed to the article and approved the submitted version.

## Conflict of Interest

The authors declare that the research was conducted in the absence of any commercial or financial relationships that could be construed as a potential conflict of interest.
