# Supplementary material for: Care Professionals Manage the Future, Frail Older Persons the Past. Explaining Why Frailty Management in Primary Care Doesn't Always Work
Source: Front Med (Lausanne). 2020 Aug 28;7:489. doi: 10.3389/fmed.2020.00489 (PMC7485521; doi:10.3389/fmed.2020.00489)
Supplement: Supplementary file 1 [file Data_Sheet_1.pdf]

Supplementary Material: Interview Guides

1. Older persons

Thank you for taking the time for this interview. Explain interest in their perspective on the care they receive in the light of a recent acute health accident. Check informed consent.

Frailty

- What is it like to live at home at a high age and with physical challenges?
- How does this affect your emotional wellbeing? Do you feel calm and at ease, or sometimes sad?
- What are your experiences with care and support while with living at home at a high age?

Acute health incident reconstruction

- What happened?
- Why do you think it happened?
- Who was involved, at which moment in time?
- How did you experience the run-up to the incident?
- What kind of care or support did you receive?
- What could have been improved in the run-up to the incident?
- What went well in the run-up?
- Additional questions: Examples? What do you mean with that? Could you elaborate on that?

PAIEC

Did you discuss with the family doctor or practice nurse in the past six months… (if so, how did this go and what do you think about it?)

Patient activation and contextual information

- …Your ideas and expectations for a plan for care and support?
- …Different possibilities for care and support?
- …Problems with medication or side-effects?
- …Experiences with care and support or problems with it?
- …Lifestyle matters, information about how to stay healthy or improve the patient's health? (e.g. smoking, movement and food)
- …Your values and norms?

Goal setting and problem solving

- …How your own actions or behavior influence your health?
- …Which goals you would like to achieve regarding your health?
- …Support in setting up specific goals to deal with the consequences of aging?
- …Information about plans for care and support?
- …Help with applying the care plan in your daily life?
- …Helped with plans in case your health will worsen?
- …How the consequences of aging influence your life?

Coordination and follow up

- …Encouragement for courses or group activities to help you to deal better with the consequences of aging?
- …How you are doing after a visit to or by the care professional, or after a (group) activity?
- …Encouraged to take part in activities in your neighborhood that could help you?
- …Referred to a professional such as a physiotherapist or social worker, or a to a group activity?
- …Why a visit to a care or social professional or group activity will be important for you?
- …How your visits to a professional or participation in a group activity went?

Additional questions for all topics:

- What is your view on this aspect of care? Why? Example?
- To what extent is this important to you? Why (not)? Example?
- Does this type of care contribute to your self-management capacities?
- Does this type of care contribute to your feeling of being in control of care decision making?
- Does this type of care contribute to your quality of life?

2. Care professionals

Thank you for taking the time for this interview. Explain interest in their perspective on the care they receive in the light of a recent acute health accident. Check informed consent.

Frailty

- Why do you consider this patient to be frail? How do you determine frailty?
- What are your experiences with care and support for older people living at home?
- Additional questions: how does this work? Could you elaborate on that? Examples? Collaboration with other professionals?

Acute health incident reconstructions (for all 9 cases)

- What happened?
- Why do you think it happened?
- Who was involved, at which moment in time?
- How did you experience the run-up to the incident?
- What kind of care or support did you deliver or organize?
- What could have been improved in the run-up to the incident?
- What went well in the run-up?
- Additional questions: Examples? What do you mean with that? Could you elaborate on that?

PAIEC

Did you discuss with the patient in the past six months…

Patient activation and contextual information:

- …Patient's ideas and expectations for a plan for care and support?
- …Different possibilities for care and support?
- …Patient's experiences with medication or side-effects?
- …Patient's experiences with care and support or problems with it?
- …Lifestyle matters, information about how to stay healthy or improve the patient's health? (e.g. smoking, movement and food)
- …Patient's values and norms?

Goal setting and problem solving

- …How patients' own actions or behavior influence their health?
- …Which goals patients would like to achieve regarding their health?
- …Support in setting up specific goals to deal with the consequences of aging?
- …Information about plans for care and support?
- …Help with applying the care plan in their daily lives?

- …Helped with plans in case their health will worsen?
- …How the consequences of aging influence their lives?

Coordination and follow up

- …Encouragement for courses or group activities to help you to deal better with the consequences of aging?
- …How patients are doing after a visit to or by the care professional, or after a (group) activity?
- …Encouraged to take part in activities in their neighborhood that could help them?
- …Referred to a professional such as a physiotherapist or social worker, or a to a group activity?
- …Why a visit to a care or social professional or group activity will be important for them?
- …How their visits to a professional or participation in a group activity went?

Additional questions for all topics:

- What is your view on this aspect of care? Why? Example?
- To what extent is this aspect of care important for this patient? Why (not)? Example?
- Does this type of care contribute to the patient's self-management capacities?
- Does this type of care contribute to the patient's feeling of being in control of care decision making?
- Does this type of care contribute to the patient's quality of life?
