# Supplementary material for: Care Professionals Manage the Future, Frail Older Persons the Past. Explaining Why Frailty Management in Primary Care Doesn't Always Work
Source: Front Med (Lausanne). 2020 Aug 28;7:489. doi: 10.3389/fmed.2020.00489 (PMC7485521; doi:10.3389/fmed.2020.00489)
Supplement: Supplementary file 2 [file Table_1.pdf]

Supplementary Material: Tables 1, 2, 3 and 4

Table 1. Overview of the nine acute health incident cases

	Overview cases acute health incidents	Interviewed respondents
**Case 1**	Fall incident	Older person 1, family doctor, practice nurse 1
**Case 2**	COPD suffocation incident	Older person 2, family doctor, practice nurse 1
**Case 3**	Fall incident	Older person 3, family doctor, practice nurse 2
**Case 4**	Fall incident	Older person 4, family doctor, practice nurse 2
**Case 5**	Fall incident	Older person 5, family doctor, practice nurse 1
**Case 6**	Fall incident	Older person 6, family doctor, practice nurse 2
**Case 7**	Fall incident	Older person 7, family doctor, practice nurse 1
**Case 8**	Fall incident	Older person 8, family doctor, practice nurse 1
**Case 9**	High blood pressure and fall incident	Older person 9, family doctor, practice nurse 1

Table 2. Characteristics older persons with frail health in cases

	Numbers
**Age**	75 - 79 (n=1)<br>80 - 84 (n=3)<br>85 - 89 (n = 1)<br>90 - 94 (n = 4)
**Sex**	Male (n=2)<br>Female (n=7)
**Marital status**	Married (n=2)<br>Widowed (n=7)
**Living situation**	Living alone, apartment (n =5)<br>Living alone, senior's apartment (n=2)<br>Living with partner, apartment (n=1)<br>Living with partner, townhouse (n=1)

Table 3. Care professional perspective

Case management practices	Examples	Factual orientation	Normative orientation
Proactive monitoring	Blood pressure screenings, weigh-ins, the timely detection of ailments.	Medical and social facts that construct frailty need to be identified and measured.	Future oriented: preventing future loss.
Proactive planning	Making a care plan with future goals, e.g., improving muscle strength, lowering blood pressure.	Medical and social facts that construct frailty need to be predicted and translated a step-wise plan to respond to future risks.	Future oriented: preventing future loss.
Multidisciplinary collaboration	Organizing multidisciplinary consultations, sharing information and tasks.	Facts from a wide variety of disciplines that construct frailty need to be detected, identified and acted upon.	Future oriented: ensuring a holistic approach to be able to prevent future loss.
Tightening the strings	Changing medications, increasing check-up frequency, including care professionals from different disciplines.	Medical and social facts that construct frailty need to be controlled.	Future oriented: preventing future loss.

Table 4. Older person perspective

Dealing-with-loss practices	Examples	Factual orientation	Normative orientation
Accepting ailments as a part of daily life	Ignoring pain or ailments; avoiding thinking about future health goals.	Medical facts that construct frailty need to be ignored, e.g., deliberately ignoring potential signs of physical loss.	Past-and-present oriented: reconciling with loss of physical capacities from the past in the present.
Putting their own situation in perspective	Comparing self to others who are worse off (e.g., sister with dementia, daughter in poor health, late friends).	Social and emotional facts need to be placed in perspective, e.g., toning down their own daily experience of loss.	Past-and-present: reconciling with all types of loss, e.g., physical capacities, social network, living situation.
Living day by day and trying to continue doing what they used to do	Not using a walker; providing informal care to partner; cooking despite becoming blind.	Medical and social facts that construct frailty need to be valued considered in relation to identity threats.	Past-and-present-oriented: maintaining their identities and lifestyles as built in the past; maintaining a feeling of self and purpose in life.
Grieving	Reminiscing about lost loved ones, lost social relations, meaningful activities that are no longer possible, e.g., traveling, reading, ice skating.	Social and emotional facts that construct frailty need explicit acknowledgement and attention, e.g., on one's daily experience with loss.	Past-and-present-oriented: being attentive to present grief caused by past loss.
